# Endovascular Management of Post-Dissection Thoracoabdominal Aortic Aneurysms After the Acute Phase: Mid-Term Outcomes of F/BEVAR

**DOI:** 10.3390/jcm15176739

**Published:** 2026-08-30

**Authors:** Victor Bilman, Natalie Noam, Lihi Cohen, Moshe Halak, Daniel Silverberg

**Affiliations:** 1 The Department of Vascular Surgery, The Chaim Sheba Medical Center, Tel-HaShomer, Ramat Gan 5266202, Israel; victor.bilman@sheba.health.gov.il (V.B.); natalie.noam@sheba.health.gov.il (N.N.); lihi.cohen2@sheba.health.gov.il (L.C.); moshe.halak@sheba.health.gov.il (M.H.); 2 Gray Faculty of Medical & Health Sciences, Tel Aviv University, Tel Aviv 6997801, Israel

**Keywords:** acute aortic dissection, stent graft, false lumen expansion, thoracic endovascular aortic repair

## Abstract

**Background/Objectives**: Not all patients with acute aortic dissection undergo endovascular repair at initial presentation, and surgical or endovascular interventions are often required for late aneurysmal degeneration. This study aims to report the mid-term outcomes of fenestrated/branched endovascular aortic repair (F/BEVAR) of post-dissection thoracoabdominal aortic aneurysms (PDTAAAs) and to compare them with the outcomes of patients treated for degenerative thoracoabdominal aortic aneurysms (DTAAAs). **Methods**: A single-center retrospective analysis was performed, including all consecutive patients who underwent F/BEVAR for TAAAs between 2020 and 2025. A total of 95 patients were identified (18 PDTAAAs and 77 DTAAAs). All patients underwent elective repair; ruptured TAAAs were excluded. A comparative analysis was performed between the PDTAAA and DTAAA groups. **Results**: During the study period, a total of 95 patients who underwent F/BEVAR for TAAAs (mean age 66 ± 10 years, males *n* = 68, 72%) were identified. Eighteen patients (males *n* = 14, 78%) underwent F/BEVAR for PDTAAAs, and 77 (males *n* = 54, 78%) for DTAAAs. The patients in the post-dissection group were younger (62 ± 14 years vs. 70 ± 8 years; *p* < 0.01). Aneurysms in the PDTAAA group more commonly involved the proximal descending aorta (Crawford type 1 and 2 TAAA: *n* = 12/18, 67% vs. *n* = 14/77, 18%; *p* < 0.01). Aortic arch debranching procedures were performed at a higher rate in the PDTAAA group (8/18, 44% vs. 8/77, 10%; *p* < 0.01). No mortality within 30 days occurred in the post-dissection group but it did occur in 2/77 (3%) patients in the degenerative group (*p* = 0.68). Mean follow-up was 31 months (range: 3–121 months). Freedom from reintervention was similar between the two groups (55% vs. 60%, non-significant). Overall survival was significantly higher in the PDTAAA group (90% vs. 60%; *p* = 0.021). **Conclusions**: F/BEVAR is technically feasible for PDTAAAs. Technical success and reintervention rates do not differ between post-dissection and degenerative TAAAs; however, overall survival is higher in patients with post-dissection aneurysms.

## 1. Introduction

Acute thoraco-abdominal aortic dissection is a life-threatening condition that, depending on its clinical presentation (complicated vs. uncomplicated), is managed either conservatively or by surgical or endovascular intervention [1]. Regardless of the initial treatment strategy, these patients require strict imaging follow-up, as late adverse aortic remodeling occurs in up to 55% of medically managed patients at 5 years [1,2].

Chronic post-dissection thoracoabdominal aortic aneurysm (PDTAAA) is a unique and challenging sequela of aortic dissection, characterized by a complex aortic architecture comprising both a true lumen (TL) and a false lumen (FL). The persistent pressurization and flow within the FL can impair favorable aortic remodeling and fuel ongoing aneurysmal degeneration, increasing the risk of late rupture and adverse outcomes. Unlike degenerative aortic aneurysms, post-dissection aneurysms are marked by residual intimal flaps and multiple re-entry tears, profoundly influencing therapeutic approaches and success rates. Traditional open surgical repair for these aneurysms is associated with high morbidity and notable perioperative mortality, with in-hospital death rates reported as 6 to 20% [3,4,5]. According to a systematic review, pooled outcomes included a mortality rate of 8%, with dialysis occurring in 8% of patients, stroke in 6%, and SCI in 4% [6].

Endovascular aneurysm repair (EVAR) using fenestrated or branched endografts (F/BEVAR) has increasingly been adopted as a less invasive alternative to open surgical repair for these high-risk patients [1,7,8,9,10,11]. Compared with open repair, the endovascular approach has been linked to reduced intraoperative blood loss and transfusion needs, lower rates of postoperative respiratory failure, tracheostomy, and dialysis, as well as a shorter length of hospital stay [10]. In a multicenter study, F/BEVAR for PDTAAA was associated with a 30-day spinal cord injury rate of 6% (paraplegia in 2%) and a 30-day mortality rate of 2% [10]. The role of thoracic endovascular aortic repair (TEVAR) alone in patients with PDTAAA remains debated as the available evidence suggests that it fails to achieve favorable aortic remodeling in the thoracoabdominal segment and is associated with high reintervention rates during follow-up [1,12]. The principal goal is to promote FL thrombosis and induce favorable aortic remodeling while preserving flow to vital branch vessels arising from either the TL or FL. There have been several reports of F/BEVAR for these aneurysms; however, there is conflicting data regarding perioperative mortality, spinal cord ischemia (SCI), and reintervention rates [7,8,9,10,11].

Fenestrated and branched endovascular repair is increasingly becoming the treatment of choice for degenerative thoracoabdominal aortic aneurysms (DTAAAs) as well as for post-dissection aneurysms; the two entities, however, differ substantially in both pathophysiology and epidemiology [10]. This study therefore aims to report the mid-term outcomes of F/BEVAR for post-dissection thoracoabdominal aortic aneurysms and to compare them with those of patients treated for DTAAAs.

## 2. Materials and Methods

This is a single-center retrospective analysis of all consecutive patients who underwent F/BEVAR at our institution for TAAAs between 2020 and 2025. A total of 95 patients were identified and divided into two groups: Group 1 comprised 18 patients treated for PDTAAAs, and Group 2 comprised 77 patients treated for DTAAAs. Data collected included patient demographics, aortic anatomical features, procedural data, aortic remodeling, FL thrombosis, residual endoleaks, reinterventions, and survival. This study was approved by our Institutional Review Board (SMC 2209-25) and was conducted in accordance with the Declaration of Helsinki. Given the retrospective nature of the study, the requirement for informed consent was waived by the IRB.

### 2.1. Patient Cohort

All patients that underwent elective TAAAs were included in this study. Patients with ruptured TAAAs were excluded. Patients with PDTAAAs were identified based on computed tomography angiography (CTA) findings documenting a dissection flap originating from the thoracic aorta and extending distally. Aneurysms with focal dissections were not considered TAAAs. Aneurysms were classified using Crawford’s classification [13].

### 2.2. Debranching Procedures

Patients underwent an aortic arch debranching procedure to create a proximal landing zone in the healthy aorta of at least 20 mm in length. These included either surgical debranching (aorto-bicarotid bypass, carotid-carotid-subclavian bypass, and carotid-carotid bypasses) or endovascular debranching using a thoracic branch endoprosthesis.

### 2.3. Stent Graft Selection

The decision on which stent graft to use, whether a fenestrated or branched device (inner or outer branches), was based on several criteria. The choice between fenestrated and branched components for each target visceral vessel was individualized according to anatomical characteristics assessed on preoperative CTA reconstructions. Fenestrations were preferentially selected in the presence of a narrow true lumen at the level of the target vessel, a configuration more frequently encountered in post-dissection anatomy, given their lower profile and shorter bridging stent requirement within a confined true lumen working space. Fenestrations were similarly favored when the gap distance between the aortic wall and the visceral vessel origin was short, permitting stable, near-orthogonal alignment between the fenestration and the target vessel ostium. Conversely, branched components were preferred in cases with a wider true lumen or aortic diameter at the target vessel level and a longer gap distance and/or greater target vessel angulation or mobility, as the branch configuration is better able to bridge distance and accommodate angulation without compromising apposition. These anatomical criteria were applied consistently by the treating physicians during device planning. 

Bridging stents were exclusively balloon-expandable stent grafts, either Bentley (Bentley InnoMed GmbH, Hechingen, Germany) or VBX (W. L. Gore & Associates, Flagstaff, AZ, USA). Self-expandable endografts were not used.

### 2.4. Spinal Cord Protection

In patients with extensive aneurysmal disease who were considered high risk for SCI, the procedures were staged with TEVAR placed in the first stage, followed by F/BEVAR 8 to 12 weeks after. Preoperative spinal drains were selectively placed in patients who were considered high risk for SCI. These included patients with coverage of >20 cm of the thoracic aorta, occluded internal iliac arteries, compromised flow to one or both vertebral arteries, and previous or simultaneous treatment of the abdominal aorta. The cerebrospinal fluid (CSF) pressure threshold was set at 10 mmHg. Spinal drains were removed 3 days after the surgery if no signs of SCI were observed. Additional protective measures included maintaining mean arterial pressure ≥ 90 mmHg and post-operative hemoglobin concentration ≥ 10 g/dL.

### 2.5. Procedural Data

The procedure, including the selection of the endograft type, was planned by 2 senior vascular surgeons. Once a specific endograft was chosen, the CTA data was transferred to the planning center of the manufacturing company to evaluate feasibility and proceed with the planning and manufacturing. In patients with PDTAAA, the preoperative CTA axial images were carefully examined to determine flow within the TL and FL, which visceral vessels originated from the FL and which from the TL, and to recognize the presence of fenestrations within the dissection flap that could provide access to the target vessels for the purpose of cannulation. All procedures were performed in a hybrid endovascular room with a fixed imaging unit.

The stent grafts were deployed in a standard fashion. If a fenestration in the dissection flap was present and the target vessel could be accessed through it, cannulation was performed with a wire and catheter, followed by placement of a bridging stent. In cases where there was no fenestration present, and it was not possible to cross the dissection flap into the FL, an attempt was made to perforate the dissection flap with the back of a wire supported by a guiding catheter. In rare cases, perforation of the dissection flap was obtained using the electrified wire technique, as described by others [14]. Technical success was defined as the deployment of the aortic endograft and all intended side-branch components.

### 2.6. Statistical Analysis

Categorical variables are presented as percentages, whereas continuous variables are reported as mean ± standard deviation. Continuous and categorical variables were analyzed using Student’s *t*-test and χ^2^ test, as appropriate. Statistical significance was set at *p* < 0.05, and all tests were two-sided. Follow-up duration was calculated for each patient from the index procedure to the last documented clinical or imaging contact, or to death, and is reported separately for the dissection and non-dissection groups as median with interquartile range (IQR) and full range. Because follow-up was not uniform, time-dependent outcomes (freedom from reintervention and survival) were analyzed using the Kaplan–Meier method, in which patients are censored at their last follow-up, and groups were compared with the log-rank test. Wizard Statistics (Version 2.0.20, EvanMiller.org) and R-Studio (Version 1.1.463, RStudio, Inc, Boston, MA) software were used for data analysis.

## 3. Results

During the study period, 95 patients underwent F/BEVAR for TAAAs. The mean age was 66 ± 10 years, and 68 patients (72%) were male. Eighteen patients underwent repair for post-dissection TAAA, whereas 77 were treated for degenerative TAAA. Baseline demographics and comorbidities are detailed in Table 1. Patients in the post-dissection group were younger than those in the degenerative group (62 ± 14 years vs. 70 ± 8 years; *p* = 0.01). The post-dissection group also had a lower prevalence of coronary artery disease (22% vs. 49%; *p* = 0.024), hypertension (67% vs. 85%; *p* = 0.03), and chronic obstructive pulmonary disease (17% vs. 26%; *p* = 0.05). American Society of Anesthesiologists (ASA) classification was similar between groups (mean ASA class 3 vs. 3; *p* = 1.0).

### 3.1. Aneurysm Morphology

Aneurysm morphology is summarized in Table 2. Post-dissection TAAAs more frequently involved the proximal descending thoracic aorta (Crawford types I and II) compared with degenerative aneurysms (67% [12/18] vs. 18% [14/77]; *p* < 0.01). In contrast, degenerative aneurysms more commonly involved more extensive thoracoabdominal segments, particularly Crawford types III and V.

### 3.2. Operative Details

Operative characteristics are presented in Table 3. The mean aneurysm diameter was similar between groups (70 ± 13 mm for post-dissection vs. 68 ± 13 for degenerative aneurysms; *p* = 0.42). In the post-dissection group, 17 of 18 cases (94.4%) showed a maximum diameter ≥55 mm. The true lumen measured 21.7 ± 9.7 mm (median: 17.5 mm; range: 12–40 mm) and the false lumen measured 41.1 ± 14.4 mm (median: 45.0 mm; range: 17–58 mm), giving a true/false lumen ratio of 0.72 ± 0.64 (median: 0.35; range: 0.22–2.11). The false lumen was therefore the dominant channel in the majority of cases, with a true/false ratio <0.5 in 11 of 18 studies (61.1%).

A total of 258 target vessels were incorporated, corresponding to a mean of 2.7 branches per patient, without differences between groups (2.5 vs. 2.7 branches per patient; *p* = 0.63). In the post-dissection group, the origin of each visceral and renal vessel was classified as arising from the true lumen, the false lumen, or from both lumens (dual supply across the dissection flap). Of the 72 vessels assessed, 58 (80.6%) arose exclusively from the true lumen, 9 (12.5%) from the false lumen and 5 (6.9%) had a dual supply. Regarding involvement, the coeliac trunk (16 true lumen, 1 false lumen, 1 dual; 11.1% not solely true-lumen) and the superior mesenteric artery (15/1/2; 16.7%) were the least frequently involved, while the renal arteries were the most frequently involved (right renal: 15/3/0 (16.7%); left renal: 12/4/2 (33.3%)). All four visceral/renal vessels originated from the true lumen in 7 cases (38.9%), whereas 8 (44.4%) had one vessel and 3 (16.7%) had two vessels arising from the false lumen or with a dual supply. One patient (5.6%) had an accessory right renal artery, which originated from the true lumen.

Cannulation of visceral arteries originating from the FL was successfully achieved with a catheter and wire in 16/18 (89%) cases. Additional techniques (steerable sheath, transcatheter electrosurgical septotomy) were used in 2 cases in order to perforate the dissection flap.

Staged repair was performed more frequently in patients with post-dissection aneurysms than in those with degenerative aneurysms (61% vs. 10%; *p* < 0.01). Similarly, adjunctive debranching procedures were more common in the post-dissection group (8/18 [44%] vs. 8/77 [10%]; *p* < 0.01). Median procedural time was comparable between groups (3.1 h [range, 1.4–6.1 h] vs. 3.4 h [range, 2.5–8 h]; *p* = 0.78). Spinal drainage was used in 50% of post-dissection cases and 58% of degenerative cases (*p* = 0.47).

The types of endografts used are presented in Table 4. In the PDTAAA group, the most commonly used were patient-specific fenestrated stent grafts. The devices used in the DTAAA group were more variable, including fenestrated and branched (inner and outer) devices.

### 3.3. Early Outcomes and Follow-Up

Technical success was achieved in 17 of 18 patients (94%) in the post-dissection TAAA (PDTAAA) group and in 75 of 77 patients (97%) in the degenerative TAAA (DTAAA) group (*p* = 0.78) Table 5. Within 30 days, no cases of spinal cord ischemia occurred in the PDTAAA group, whereas 3/77 cases (4%) were observed in the DTAAA group (*p* = 0.57). No mortality within 30 days occurred in the PDTAAA group; however, 2/77 deaths (3%) occurred in the DTAAA group (*p* = 0.68). Median post-operative length of stay was 6 days [IQR: 5–10] for the overall cohort, with no significant difference between the dissection and non-dissection groups (6 [5,6,7] vs. 6 [5,6,7,8,9,10,11] days; *p* = 0.62).

The mean duration of follow-up was 31 months (range, 3–121 months). During follow-up, complete false lumen thrombosis was achieved in 54% of patients in the PDTAAA group. Residual type II endoleaks were identified in 8 patients (44%) in the PDTAAA group and in 25 patients (32%) in the DTAAA group (*p* = 0.39). Sac regression or stability was observed in 98% of the overall cohort, including all patients (100%) in the PDTAAA group and 97% in the DTAAA group (*p* = 0.44). Four branch occlusions (4%) occurred in three patients overall. In the PDTAAA group, one patient developed left renal artery branch occlusion at 5 months following the index procedure, in a target vessel incorporated with an off-the-shelf inner branch device. In the DTAAA group, two patients developed branch occlusion involving a total of three renal arteries: one patient developed right renal artery occlusion at 18 months, and a second patient developed bilateral renal artery occlusion at 24 months. Both patients in the DTAAA group had been treated with an off-the-shelf inner branch device.

Branch occlusion occurred in 1 patient (5%) in the PDTAAA group and in 3 patients (4%) in the DTAAA group (*p* = 0.86). Median follow-up was 27.2 months (IQR: 8.7–41.3; range: 0.2–141.6) in the dissection group and 15.3 months (IQR: 1.7–34.2; range 0.03–103.6) in the non-dissection group; the difference was not statistically significant (mean: 31.6 ± 33.2 vs. 22.5 ± 23.7 months, *p* = 0.29).

### 3.4. Reinterventions

**Figure 1:**
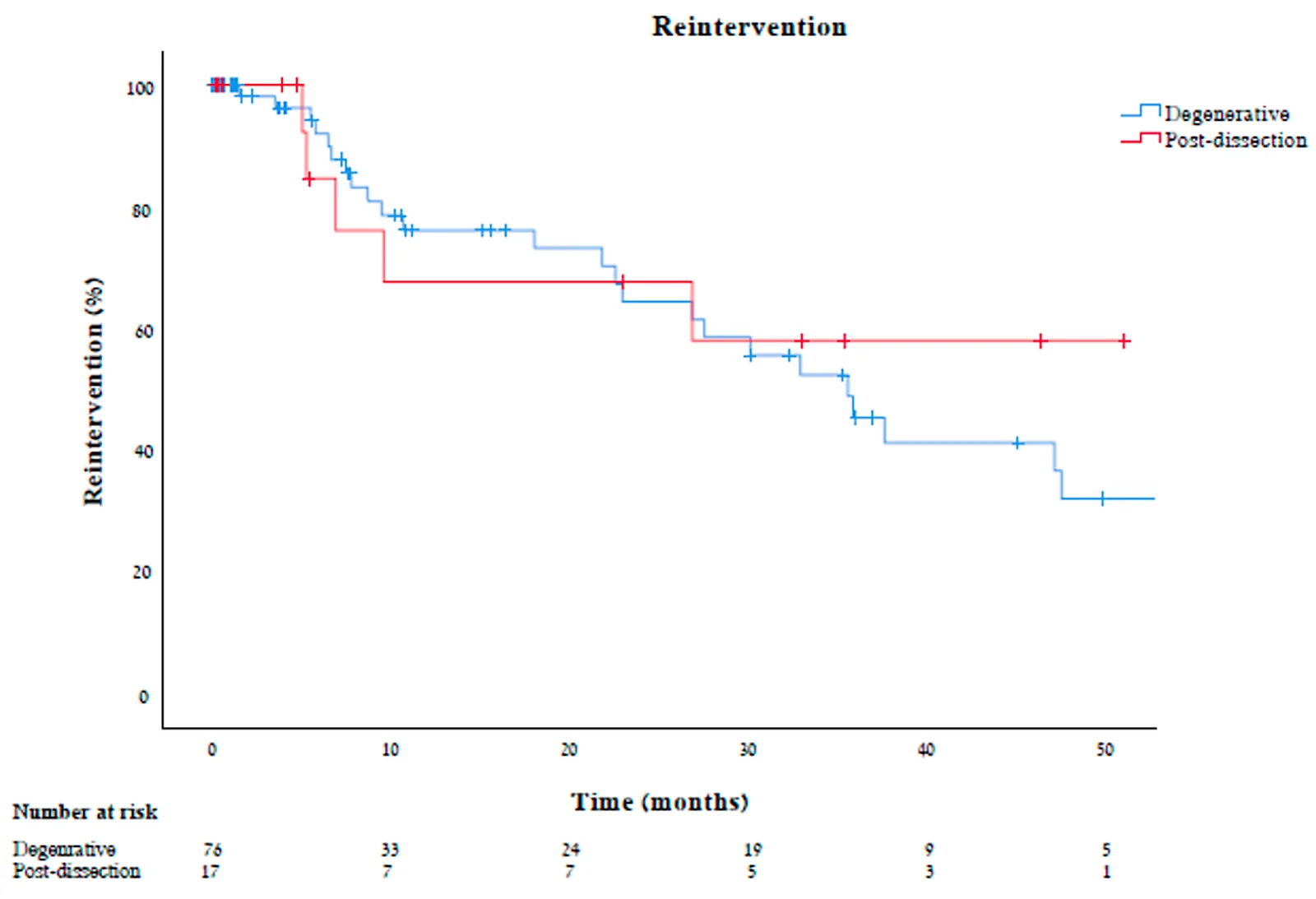
Kaplan–Meier estimates of freedom from reintervention comparing post-dissection (PDTAAA) and degenerative (DTAAA) thoracoabdominal aortic aneurysms following F/BEVAR. Freedom from reintervention at 40 months was 55% for PDTAAA and 60% for DTAAA (log-rank *p* = non-significant).

Reintervention was required in 7/18 patients (39%) in the PDTAAA group and in 29/77 patients (38%) in the DTAAA group. In the PDTAAA group, 3 reinterventions were due to branch complications, whereas 4 were performed as adjunctive procedures to treat persistent false-lumen perfusion. These included aortobifemoral bypass (*n* = 2), iliac limb extension (*n* = 1), and renal side-branch occlusion with iliorenal bypass (*n* = 1). Freedom from reintervention at 40 months was similar between groups (55% for PDTAAA vs. 60% for DTAAA; not significant) (Figure 1).

### 3.5. Survival

**Figure 2:**
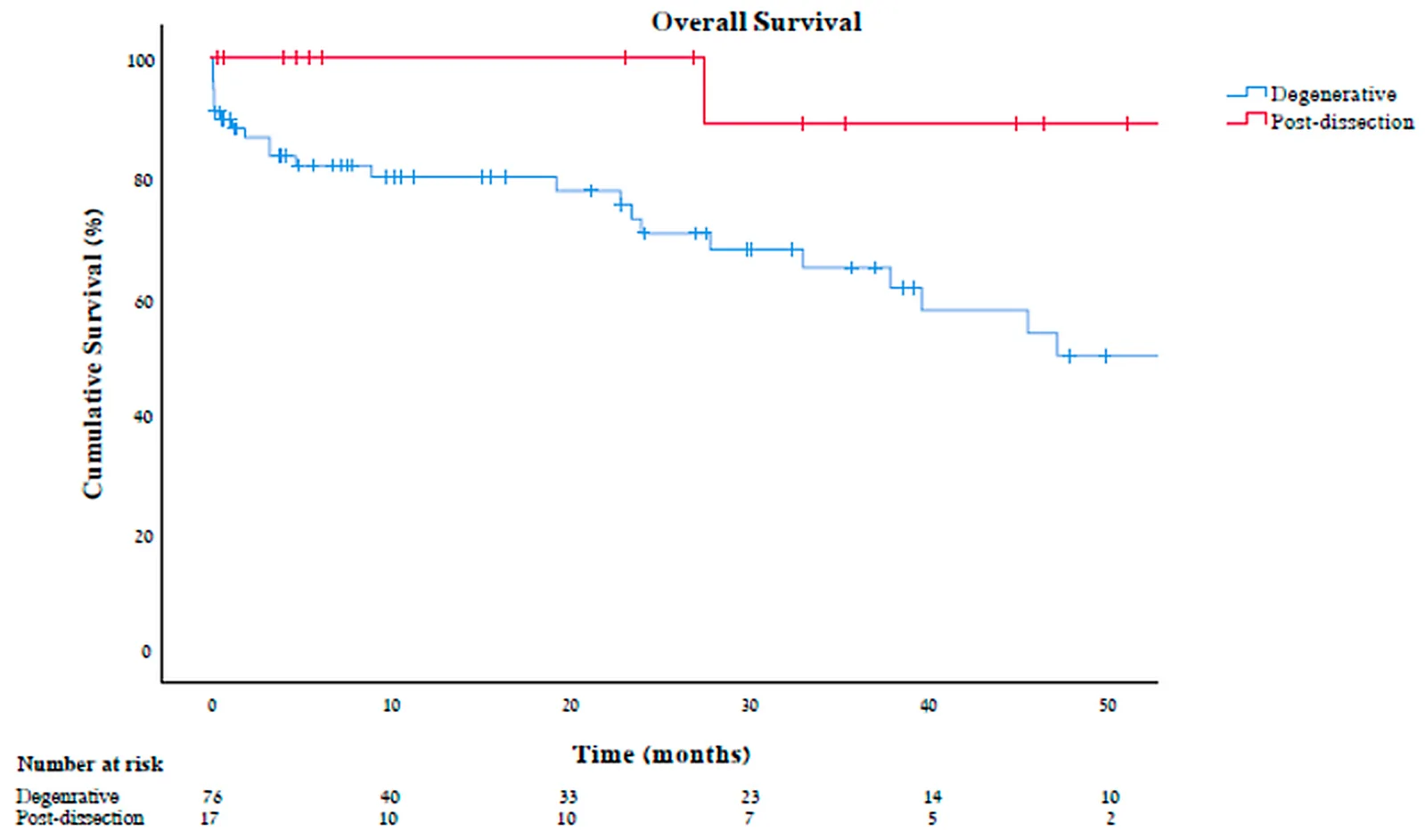
Kaplan–Meier estimates of overall survival comparing post-dissection (PDTAAA) and degenerative (DTAAA) thoracoabdominal aortic aneurysms following F/BEVAR. Overall survival at 40 months was 90% for PDTAAA and 60% for DTAAA (log-rank *p* = 0.021).

During follow-up, 10 patients in the degenerative group died, including 2 deaths related to aneurysm. In the post dissection group, 1 patient died from a non-aneurysm-related cause. Kaplan–Meier analysis demonstrated lower overall survival in the degenerative group compared with the post-dissection group at 40 months (60% vs. 90%; log-rank *p* = 0.021) (Figure 2).

## 4. Discussion

This study demonstrates that patients who undergo conservative management during the acute phase of aortic dissection may subsequently develop adverse aortic remodeling, often requiring complex endovascular reconstruction during follow-up. Our findings further highlight that, although F/BEVAR is a well-established treatment for degenerative thoracoabdominal aortic aneurysms, its application in the post-dissection setting represents a distinct undertaking—shaped by a different patient population and a fundamentally different aortic pathology.

Post-dissection TAAA repairs remain among the most challenging clinical entities for vascular surgeons performing aortic surgery. This single-center study shows that PDTAAAs can be treated safely with F/BEVAR with a high success rate and acceptable morbidity and mortality. In this study, we found that when compared to degenerative aneurysms, patients with post-dissection aneurysms were typically younger and suffered from fewer cardiovascular comorbidities. This finding correlates with that reported by Odonell et al., who found in their cohort that PDTAAA patients were a decade younger in age [15]. The mean age of a patient experiencing a type B aortic dissection typically ranges between 60 and 70 years [16]. Degenerative aneurysms result from age-related weakening of the aortic wall, often due to atherosclerosis and other chronic vascular risk factors. As such, they are more common in the elderly population and are typically diagnosed between 65 and 75 years [13].

Post-dissection TAAAs in the present study were more extensive and more frequently involved the proximal descending aorta compared with degenerative TAAAs. This likely reflects their pathogenesis, as these aneurysms typically develop as late sequelae of prior type A or type B aortic dissection. The proximal extension and overall greater aortic involvement may account for the higher rate of staged procedures performed, which aimed to reduce the risk of spinal cord ischemia. Furthermore, these anatomical characteristics explain the frequent need for aortic arch debranching to achieve an adequate proximal sealing zone. In our study, 55% of patients required some form of surgical or endovascular debranching. High rates of staged procedures and aortic arch debranching in PDTAAAs have been reported in the literature. Gorgatti et al. reported that 87% of their patients with PDTAAA presented with Crawford type I or II aneurysms [17]. Similarly, O’Donnell et al. demonstrated that PDTAAAs were more extensive (84%) than degenerative aneurysms (22%) [15]. Consequently, staged repair was performed more frequently in post-dissection aneurysms (36%) than in degenerative aneurysms (6.7%) [15,17].

There are several anatomical and technical pitfalls that are commonly encountered in post-dissection aneurysms compared to degenerative aneurysms. These differences are due to factors such as TL compression and the separation of the target vessel origin from the true or false lumen. These differences can affect the choice of endografts suitable for these procedures. Currently, there is still no consensus regarding the ideal design for a post-dissection F/BEVAR device. In our study, patients in the PDTAAA group were more likely to receive a custom-made fenestrated stent graft than those in the DTAAA group. Unlike branched devices, patient-specific fenestrated endografts manufactured by COOK (Cook Medical, Bloomington, IN, USA)have the advantage of having diameter-reducing ties along the posterior aspect of the stent. This feature allows for repositioning of the endograft within the narrow TL. The use of physician-modified endografts as the primary repair type for post-dissection aneurysms has also been described [15].

An additional challenging aspect of post-dissection aneurysm repairs is cannulating target vessels originating from the FL. This is usually achieved by identifying dissection flap tears or fenestrations adjacent to the target vessels and then cannulating the target vessel with a catheter and wire. In our series, this was achievable in 89% of the cases. In those cases where it is not feasible, it may be necessary to perforate the dissection flap and create a passage between the TL and FL. This can be done using a steerable catheter or by transcatheter electrosurgical septotomy. Despite these technical challenges, no differences were observed between the groups in technical success or procedural time. These findings are consistent with previous reports demonstrating comparable technical success rates between post-dissection and degenerative TAAAs [18].

With respect to late aortic remodeling, Arnaoutakis et al. examined remodeling, reintervention, and survival after endovascular repair of chronic type B dissection, reporting that although endovascular repair promoted favorable aortic remodeling in a substantial proportion of patients, reintervention remained common during follow-up, a finding that parallels our own reintervention rate of 39% in the post-dissection group despite favorable rates of sac stability and false lumen thrombosis [12]. Similarly, O’Donnell et al., in a study of complex endovascular treatment of post-dissection aneurysms, reported that although technical and short-term outcomes were favorable, reintervention remained frequent during longer-term follow-up, reinforcing the need for rigorous surveillance in this population [15].

The rate of reinterventions following F/BEVAR for post-dissection TAAAs, and whether it is higher than that observed in degenerative TAAAs, remains controversial in the contemporary literature. O’Donnell et al. report that three-year reinterventions were significantly higher following post-dissection repairs [15]. In another study by Tenorio et al., freedom from reintervention was similar at 14 months [10]. In our study, we found no significant differences in reintervention rates between the two groups, which may be explained by the small number of patients in each group. An interesting finding of the present study was the difference in overall survival between the two groups. Survival in the post-dissection group was superior to that of the degenerative group. The logical explanation is that the PDTAAA group was younger and had fewer comorbidities than the DTAAA group.

The present study findings are consistent with, and extend, prior multicenter experience comparing F/BEVAR outcomes between post-dissection and degenerative TAAAs [10]. In the U.S. Fenestrated and Branched Aortic Research Consortium study of 240 patients, PDTAAA patients were significantly younger and more often male, with more prior aortic repairs and larger renal and mesenteric target artery diameters compared with DTAAA patients, mirroring the younger age and lower comorbidity burden observed in our post-dissection cohort. Similarly, technical success was comparable between groups (100% for PDTAAAs and 99% for DTAAAs), consistent with our technical success rates of 94% and 97%, respectively. Regarding perioperative safety, the consortium study reported no difference in 30-day mortality (2% in post-dissection, 3% in degenerative), spinal cord injury (6% in post-dissection, 12% in degenerative), or paraplegia rates (2% in post-dissection, 7% in degenerative) between groups, findings that parallel our own low and statistically similar early complication rates. Our reintervention rates, however, were considerably higher than those reported in the consortium cohort, with reinterventions required in 40% of patients treated for PDTAAAs and 30% of those with DTAAAs, with no significant difference in freedom from reintervention at two years between groups [10]. Notably, in the multicenter series, endoleaks were significantly more frequent among post-dissection TAAA patients (76%) compared with degenerative TAAA patients (43%), a disparity we did not replicate, as residual type II endoleak rates were statistically similar between our post-dissection and degenerative groups. Regarding survival, the consortium study found no significant difference in patient survival at two years between post-dissection and degenerative TAAAs (84% vs. 72%), whereas our cohort demonstrated a significant survival advantage for post-dissection patients at 40 months, likely reflecting the markedly younger age and lower cardiovascular comorbidity burden in this subgroup [10]. Taken together, these comparisons reinforce that F/BEVAR can be performed safely in both post-dissection and degenerative TAAA populations, while highlighting that late outcomes, particularly survival and reintervention, remain influenced by the distinct baseline characteristics and anatomic complexity inherent to each pathology.

### Limitations

Some limitations must be considered. First and foremost, this study is retrospective and single-centered, which limits its generalizability. As all consecutive patients treated with F/BEVAR during the study period were included without additional selection criteria, the pronounced imbalance between the PDTAAA (*n* = 18) and DTAAA (*n* = 77) groups reflects the true relative incidence of these two pathologies in our clinical practice rather than a selection bias inherent to the study design. Nevertheless, this disproportion in sample size raises concerns about statistical power and the potential for type II errors, particularly for lower-frequency outcomes such as spinal cord ischemia and 30-day mortality, where true between-group differences may not have been detected. The smaller PDTAAA cohort also results in wider confidence intervals around survival and reintervention estimates, which should be interpreted with appropriate caution and may limit the precision with which these outcomes can be compared between groups.

Additionally, the exclusion of ruptured TAAAs precludes conclusions regarding the performance of F/BEVAR in emergency settings. The relatively short follow-up duration may not fully capture late complications, such as endoleaks or branch occlusions, or accurately reflect long-term survival and reintervention outcomes. Though overall survival was higher in the PDTAAA group, confounding variables such as age and comorbidities likely played a significant role in this difference, and the retrospective design does not allow for adjustment of these confounders through multivariable analysis given the limited number of events in the PDTAAA group.

Furthermore, several procedural factors may have influenced the outcomes independently of aneurysm etiology. There was device heterogeneity between groups, with a higher proportion of patient-specific fenestrated devices used in the PDTAAA group and a more variable mix of fenestrated and branched, off-the-shelf and patient-specific devices used in the DTAAA group; the lack of standardization in stent graft type may have influenced procedural and clinical outcomes in ways not fully captured by our comparison. Staged procedures and adjunctive debranching were also performed more frequently in the PDTAAA group, reflecting the greater anatomic complexity and more frequent proximal descending aortic involvement in this population, and may have independently affected the procedural risk, spinal cord protection, and reintervention rates. Finally, all procedures were performed by a small group of experienced operators at a single center, and operator experience and evolving procedural technique over the study period may have influenced technical success and complication rates in ways that cannot be fully accounted for in this analysis. Multicenter studies and prospective analyses with larger, more balanced cohorts should be conducted to confirm these findings, allow for more robust statistical adjustment, and ensure generalizability.

## 5. Conclusions

F/BEVAR is technically feasible among PDTAAAs. As these aneurysms are extensive and involve the proximal descending aorta, they frequently require debranching of the aortic arch to create an adequate landing zone. Technical success and reintervention rates did not differ between the post-dissection and degenerative aneurysm groups. Although overall survival was higher among patients with post-dissection aneurysms, this finding likely reflects the younger age and lower comorbidity burden of this group rather than an inherent effect of aneurysm etiology on survival and should be interpreted with appropriate caution.

## Figures and Tables

**Table 1:** Demographics and comorbidities of patients treated for post-dissection and degenerative aortic aneurysms.

Comorbidity	Overall (*n* = 95)	Post-Dissection (*n* = 18)	Degenerative (*n* = 77)	*p* Value
Males	68 (72)	14 (78)	54 (70)	0.78
Age, years	66 ± 10	62 ± 14	70 ±8	0.01
Coronary Artery Disease	42 (44)	4 (22)	38 (49)	0.024
Congestive Heart Failure	10 (11)	2 (11)	8 (10)	0.83
Hypertension	78 (82)	12 (67)	66 (85)	0.03
Diabetes Mellitus	13 (14)	3 (17)	10 (13)	0.69
Hypercholesterolemia	77 (81)	11 (61)	66 (86)	0.12
Chronic Obstructive Pulmonary Disease	23 (24)	3 (17)	20 (26)	0.05
Past Smoking	79 (83)	13 (72)	66 (86)	0.27
Current Smoking	38 (40)	8 (44)	30 (39)	0.69
Chronic Kidney Disease	17 (18)	2 (11)	15 (19)	0.48
Prior aortic surgery	42 (44)	8 (44)	34 (44)	1
ASA Classification (mean)	3	3	3	1

ASA, American Society of Anesthesiologists. Categorical variables are presented as number (%). Continuous variables are presented as mean ± SD.

**Table 2:** Aneurysm morphology of patients treated for post-dissection and degenerative aortic aneurysms.

Aneurysm Anatomy (Crawford Type)	I	II	III	IV	V
Post-dissection (*n* = 18)	10 (56%)	2 (11%)	2 (11%)	1 (5%)	3 (17%)
Degenerative (*n* = 77)	7 (9%)	7 (9%)	19 (25%)	10 (13%)	34 (44%)

Categorical variables are presented as number (%).

**Table 3:** Operative data.

Variable	Overall (*n* = 95)	Post-Dissection (*n* = 18)	Degenerative (*n* = 77)	*p* Value
Aneurysm diameter, mm	69 ± 10.9	70 ± 13	68 ± 13	0.42
Number of branches, total	258 (2.7/patient)	46 (2.5/patient)	212 (2.7/patient)	0.63
Staged procedures	19 (20)	11 (61)	8 (10)	<0.01
Debranching	16 (17)	8 (44)	8 (10)	<0.01
Procedural time, hours	3.3 (range 1.4–8)	3.1 (range 1.4–6.1)	3.4 (range 2.5–8)	0.78
Spinal drain	54 (57)	9 (50)	45 (58)	0.47

Categorical variables are presented as number (%). Continuous variables are presented as mean ± SD.

**Table 4:** Types of endografts used for treatment of post-dissection and degenerative aortic aneurysms.

Stent Graft	Overall (*n* = 95)	Post-Dissection (*n* = 18)	Degenerative (*n* = 77)
COOK, Fenestrated (patient specific)	33 (35)	11 (61)	21 (27)
COOK, T-Branch (off the shelf)	3 (3)	0 (0)	3 (4)
Artivion *, EXTRA (patient specific)	35 (37)	3 (17)	32 (42)
Artivion *, ENSIDE (off the shelf)	25 (26)	4 (22)	21 (27)

Categorical variables are presented as number (%). * Artivion, Hechingen, Germany.

**Table 5:** Surgical outcomes following fenestrated and branched endovascular aneurysm repair (F/BEVAR) for post-dissection and degenerative aortic aneurysms.

Variable	Overall (*n* = 95)	Post-Dissection (*n* = 18)	Degenerative (*n* = 77)	*p* Value
Technical success	92 (97)	17 (94)	75 (97)	0.78
30-day mortality	2 (2)	0	2 (3)	0.68
Myocardial infarction	1 (1)	1 (6)	0	0.11
Acute kidney injury	7 (7)	2 (11)	5 (6)	0.64
Cerebrovascular event	2 (2)	1 (6)	1(1)	0.34
Spinal cord ischemia	3 (3)	0	3 (4)	0.57
Length of stay, days	6 [IQR 5–10]	6 [IQR 5–7]	6 [IQR 5–11]	0.62
**Follow up**				
Sac regression/stable	93 (98)	18 (100)	75 (97)	0.44
False lumen thrombosis	NA	14 (78)	NA	NA
Residual type II endoleaks	33 (35)	8 (44)	25 (32)	0.39
Adjunctive false lumen intervention	NA	4 (22)	NA	NA
Branch occlusion, per branch	4 (4)	1 (5)	3 (4)	0.86

NA, not applicable. IQR, interquartile range. Categorical variables are presented as number (%). Continuous variables are presented as median [interquartile range].

## Data Availability

The data are not publicly available due to patient privacy restrictions but are available from the corresponding author upon reasonable request and institutional approval.

## Abbreviations

The following abbreviations are used in this manuscript:

PDTAAA	Post-dissection thoracoabdominal aortic aneurysm
DTAAA	Degenerative thoracoabdominal aortic aneurysm
TAAA	Thoracoabdominal aortic aneurysm
TL	True lumen
FL	False lumen
EVAR	Endovascular aneurysm repair
F/BEVAR	Fenestrated or branched endovascular aneurysm repair
CTA	Computed tomography angiography
SCI	Spinal cord ischemia
CSF	Cerebrospinal fluid
ASA	American Society of Anesthesiologists
SD	Standard Deviation
NA	Not applicable
SMC	Sheba Medical Center
